# Development and Characterization of Niaprazine-Loaded Xanthan Gum-Based Gel for Oral Administration

**DOI:** 10.3390/gels11020101

**Published:** 2025-02-01

**Authors:** Elena Giuliano, Emanuela Longo, Agnese Gagliardi, Silvia Costa, Federica Squillace, Silvia Voci, Mario Verdiglione, Donato Cosco

**Affiliations:** 1 Department of Health Sciences, University of Catanzaro “Magna Græcia”, Campus Universitario “S. Venuta”, I-88100 Catanzaro, Italy; elena.giuliano@unicz.it (E.G.); emanuela.longo@unicz.it (E.L.); gagliardi@unicz.it (A.G.); silvia.voci@unicz.it (S.V.); 2 Apotiga Laboratory, Farmacia Europea, Via Milano, 24/A, I-88100 Catanzaro, Italy; costa.farmaciaeuropea@gmail.com (S.C.); squillace.farmaciaeuropea@gmail.com (F.S.)

**Keywords:** niaprazine, xanthan gum gels, pediatric insomnia, magistral formulations

## Abstract

Niaprazine is a sedative-hypnotic drug initially developed as an antihistamine and used for its notable sedative effects, particularly in children. Following its withdrawal from the market by the producer, the drug has been administered as magistral formulations available in syrup form, but there are several important disadvantages to this, including instability, taste issues, lack of controlled release, and the potential for unreliable dosing due to incomplete swallowing. There is also an increased risk of dental caries, as well as the fact that these formulations are not suitable for children who suffer from diabetes. The purpose of the current investigation is to prepare and characterize xanthan gum-based gels for the oral administration of niaprazine. Niaprazine gels appear as transparent-whiteish, non-sticky substances, with the drug uniformly dispersed throughout the systems. They are also stable over time. Dynamic rheology revealed their advantageous shear-thinning properties, which enable the formulation to be flexibly dosed orally through administration via syringe. During experimentation, the evaluation of the mucoadhesion features and the in vitro drug release profile were also performed. The results demonstrate that the formulation may represent an alternative to niaprazine syrup, allowing easy preparation, administration, and increased compliance in various categories of patients, including pediatric.

## 1. Introduction

Currently, the large-scale manufacture of medications for the worldwide market is standard practice [1]. However, industrial medical products are sometimes unavailable or may not be the most suitable option for the treatment of specific patients or pathological conditions [1,2]. These include the well-known orphan diseases, as well as neglected dosages or administration routes, as well as patients’ sensitivity to excipients [1,2]. Magistral and officinal pharmaceutical formulations prepared in community and hospital pharmacies can be an effective alternative for patients when industrial medicinal products are unavailable on the market [2,3].

The indication “magistral preparation” describes “any medicinal product prepared in a pharmacy in accordance with a medical prescription for an individual patient” [3]. It is a practical way to personalize the treatments of patients with specific needs and to obtain pharmaceutical formulations when no commercial products are available [4].

This practice is commonly employed in pediatric pharmacy [4]. Many of the medicines prescribed and administered to babies and children lack marketing authorization or product license [4].

**Figure 1:**
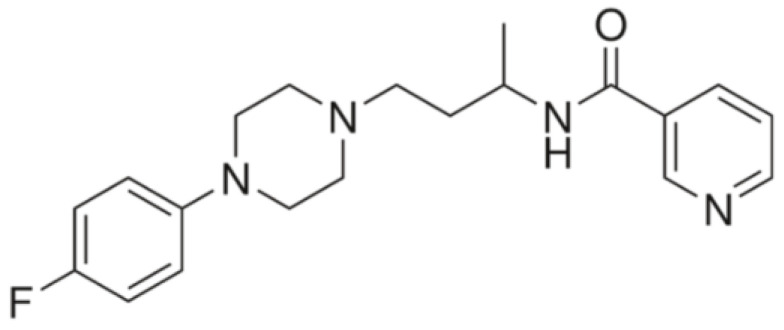
Chemical Structure of N-[3-[4-(p-fluorophenyl)- 1-piperazinyl]- l-methylpropyl]-3 pyridinecarboxamide (niaprazine).

Niaprazine (N-[3-[4-(p-fluorophenyl)- 1-piperazinyl]- l-methylpropyl]-3 pyridinecarboxamide) is a sedative-hypnotic drug belonging to the family of phenylpiperazine (Figure 1) [5].

Since the early 1970s, it has been used under the brand name Nopron^®^ (Laboratoires Genopharm, Saint-Thibault-des-Vignes, France) for treating sleep disturbances, especially in children and adolescents [5,6,7,8].

The substance is also a classic example of a repositioned drug. Drug repositioning involves molecules that already have marketing authorization for specific therapeutic indications, thereby having well-known safety profiles recognized by regulatory agencies and scientific communities [4].

In fact, niaprazine was initially developed as an antihistamine and anticholinergic drug. Unfortunately, it was also associated with a strong sedative effect, similar to many first-generation antihistamines [8,9,10].

Nopron^®^ syrup was the only medicinal containing niaprazine available on the market, exclusively in France and Italy, until 2012, when the manufacturing company chose not to renew its marketing authorization. This decision was motivated by serious issues related to Good Manufacturing Practices, such as difficulties in ensuring compliance regarding the production, exportation, and importation of the drug, which had been identified during inspection checks [https://www.aifa.gov.it (accessed on 15 January 2025)].

Since niaprazine is no longer on the market for reasons unrelated to usage risks, it can be prepared as a magistral formulation under medical prescription. It is usually administered orally as syrup at a dosage of 1 mg/kg, preferably 30 min before bedtime [11].

However, the use of a syrup-based formulation containing niaprazine may be open to criticism because of various factors, including chemical and physical instability, taste issues, lack of controlled release properties, and unreliable dosing because of incomplete swallowing [12]. In addition, conventional syrups contain a high amount of sucrose (66.5% *w*/*w*), which has been associated with a major risk of dental caries. This risk is particularly significant if it is administered at bedtime because of the decreased protective buffering and cleansing effect of saliva due to a lower nocturnal salivary flow rate [13]. Moreover, they should certainly be avoided for pediatric patients suffering from diabetes and hereditary fructose intolerance [14].

Oral gels should be a feasible alternative for addressing these issues. Gel formulations have the advantage of preventing drug precipitation due to their high viscosity as compared to syrups. Moreover, shear-thinning systems allow flexible dosing because of their easy flow upon application of suitable stress, such as withdrawal by syringe [15].

A wide range of natural and synthetic polymers have been employed for the development of various oral formulations. In particular, polysaccharides derived from renewable resources such as plants, seaweeds, and bacterial fermentation are nontoxic, biocompatible, biodegradable, and exhibit bio-adhesive properties, making them ideal as main components of drug delivery systems [16].

Polysaccharides, characterized by a high affinity for water and the ability to significantly increase the viscosity of the solutions even at low concentrations, are commonly indicated as gums [17]. Among them, xanthan gum (XG) was widely used in the past decades due to its approval as a food additive by the US Food Drug and Administration (FDA) in 1969 [18]. XG is an exopolysaccharide that is mostly produced by aerobic fermentation of simple sugars by the Xanthomonas campestris bacterium. It is used in the food, cosmetic, and pharmaceutical industries as an emulsifier, stabilizer, and thickener [19]. It improves the stability and viscosity of oral gels, ensuring they maintain their consistency over time [19]. Moreover, XG exhibits excellent resistance to enzymatic degradation and demonstrates great stability across a broad range of temperatures, pH levels, and salt concentrations [17].

Considering the aforementioned aspects, XG was chosen as the gelling agent in the present study to develop a patient-friendly oral gel containing niaprazine.

The dynamic and passive rheological features, macroscopic properties, stability, and in vitro drug release profiles of the formulations were evaluated.

## 2. Results and Discussion

### 2.1. Stability of Niaprazine-Loaded Xanthan Gum Gels

Magistral compounding is a practice carried out by pharmacists that produces appropriate formulations when there are none commercially available, are unlicensed, or are unavailable in age-specific dosage forms [20]. As compared to industrial medicinal products, these preparations may be characterized by some drawbacks, especially in terms of stability [2].

Therefore, initially, the evaluation of the physical stability profiles of niaprazine-loaded gels was assessed.

A Turbiscan^®^ Lab Expert device (Formulaction, L’Union, France) was used to observe physical changes in the formulations over time at various temperatures. The instrument allows the detection of instability phenomena such as sedimentation, migration, and/or flocculation [21]. The analyses were carried out at 25 °C (room/storage temperature) and 37 °C (body temperature) and reported as the Turbiscan Stability Index, a parameter that correlates the backscattered and transmitted light in the sample. Generally, a stable formulation is characterized by low variation of the Turbiscan Stability Index (TSI) values [22].

**Figure 2:**
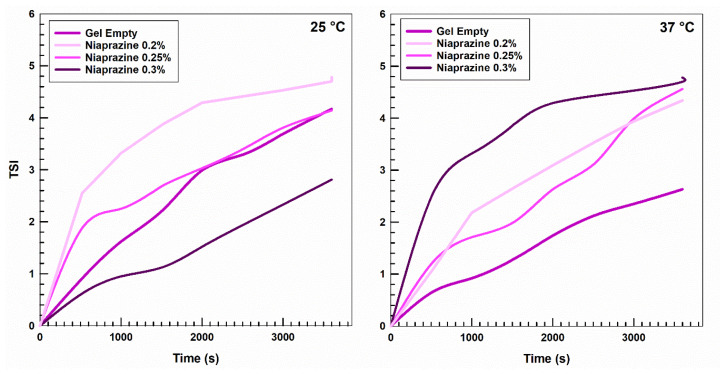
Turbiscan stability index (TSI) of niaprazine-loaded xanthan gum gels as an empty formulation or containing the drug (0.20–0.30% *w*/*v*) as a function of temperature and incubation time. The result was a representative experiment of three independent experiments.

**Figure 3:**
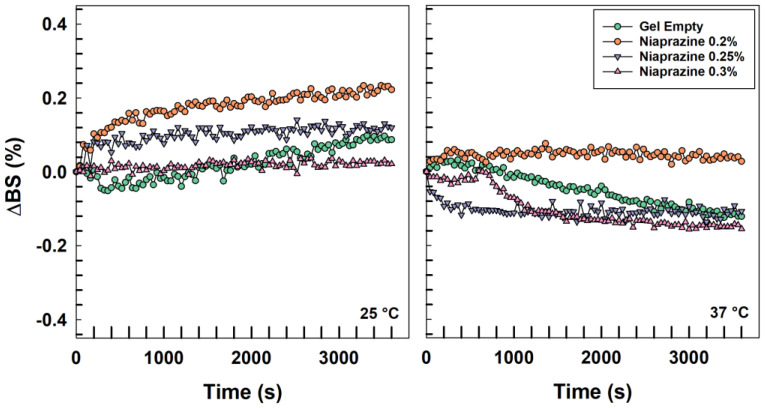
Mean values of ΔBS of niaprazine-loaded xanthan gum gel as an empty formulation or containing the drug (0.2–0.3% *w*/*v*) as a function of temperature and incubation time.

As shown in Figure 2, the presence of the sedative drug in the XG-based gels did not cause strong variations in the TSI profiles of the systems with values less than 5, showing the absence of adverse phenomena, as was also demonstrated by the ∆BS profiles close to the baseline (Figure 3 and Figure S1. In fact, variations in the backscattering profiles of samples within an interval of ±2% are not considered indicative of instability [23].

### 2.2. Physical Features

Physical observation of the pharmaceutical formulations is important to ensure patient compliance. The niaprazine gels were white-transparent, non-sticky, of acceptable consistency, and with no presence of grittiness (Figure S2 and Video S1).

No syneresis was noticed within the stability period. This phenomenon consists of the separation of water from the formulation and is a common problem associated with gel systems during their storage, especially when a lower concentration of gelling agent is employed [15].

Table S1 shows the results obtained from the homogeneity test of niaprazine 0.3% *w*/*v*-loaded gels collected at different locations within the formulation. The average value across all three locations was around 100%, demonstrating the uniform distribution of the drug in the formulation [15].

Regarding pH, the values of the developed formulations were between 5.5 (empty gel) and 6.5 (systems containing niaprazine 0.2–0.3% *w*/*v*), a range suitable for oral intake [24].

### 2.3. Rheological and Micro-Rheological Characterization

XG is a biocompatible and biodegradable anionic polysaccharide available on the market and obtained by bacterial fermentation [25]. It is widely used for its thickening and stabilizing effect thanks to its tendency to form a gel structure in aqueous solutions [25]. XG is made up of a linear β-(1-4)-D-glucose backbone with a trisaccharide side chain consisting of β-D-mannose-(1,4)-β-D-glucuronic acid-(1,2)-α-D-mannose on every glucose residue (Figure S3A) [26].

The powder is usually characterized by the formation of lumps when added to water. Therefore, it plays a crucial role in the final viscosity of the proposed formulations; in fact, inadequate dispersion and hydration can cause agglomerates in the system, decreasing the efficacy of the thickening effect.

In aqueous solution, the polysaccharide adopts two conformations as a function of temperature. At higher temperatures, XG exists as a disordered and flexible coil structure, while below 40–50 °C, it is characterized by an ordered double helical strand structure (Figure S3B) [27]. The double helixes form a three-dimensional network, thus conferring a weak gel-like behavior and shear-thinning properties to XG solutions under stress [26].

Viscometry studies were carried out at 25 °C to simulate storage conditions and at 37 °C, corresponding to normal body temperature.

**Figure 4:**
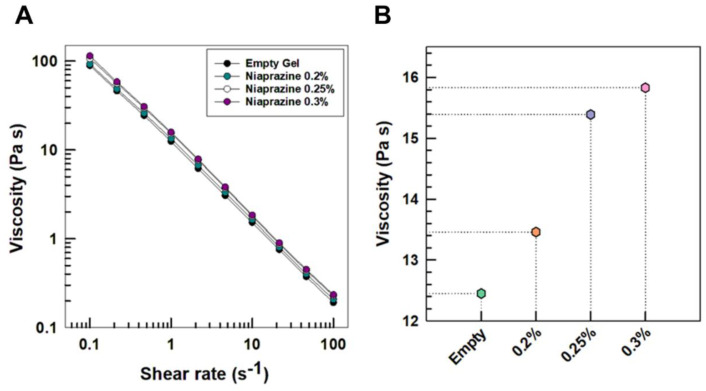
(**A**) Steady-state shear viscosity of xanthan gum gels as a function of shear rate (**A**) and drug concentration measured at constant shear rate (1 s^−1^) (**B**). Analyses were performed at 25 °C.

Flow curves, reported in Figure 4, showed the typical shear-thinning behavior of XG solutions over the entire range of shear rates (panel A), evidencing a slight influence of drug concentration on the viscosity of the systems (panel B).

The phenomenon of shear-thinning behavior is related to the orientation of the macromolecules along the streamline of the flow. At low shear rate values, the polysaccharide forms aggregates, while high viscosity is due to the large flow resistance. Increasing the shear rate destroys aggregates, and the dispersing molecules arrange themselves along the flow direction, thus reducing the local drag and, as a consequence, the apparent viscosity [27]. This behavior is classically described in the literature as a “weak gel” [28].

The rheological studies performed at 37 °C confirmed the same trend observed at 25 °C, with only a negligible decrease in the viscosity.

These results showed a useful behavior of the proposed formulation for the administration of niaprazine; that is, the gels have high viscosity at rest during storage, preventing drug precipitation, but their viscosity decreases upon application of a stress factor, such as withdrawal with a syringe, thus allowing flexible dosing as a function of body weight [15].

Moreover, these rheological features could simplify and improve the compliance of pediatric patients with difficulty swallowing [29,30].

**Figure 5:**
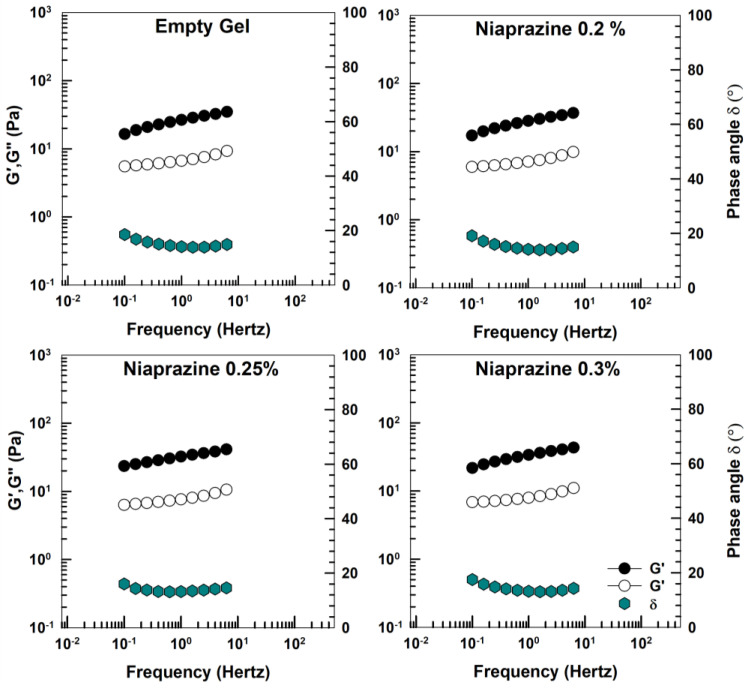
Elastic modulus (G′), viscous modulus (G″), and phase angle (δ) of niaprazine-loaded gels as a function of frequency. The analysis was performed at 25 °C.

Figure 5 shows the storage (G′) and loss (G″) moduli of XG gels acquired in the linear viscoelastic region. For all samples, G′ exceed G″ values in the measured frequency range, confirming their weak gel-like behavior [26]. At the same time, phase angle values were below 20°. The increase of temperature to 37 °C did not modify the mechanical spectra of the systems (data not shown).

The results are in agreement with those previously reported by other authors regarding XG-based gel formulations [26,31,32,33].

The micro-rheology of niaprazine gels was evaluated using the Rheolaser^®^ Master micro-rheometer. This device employs an optical measurement technique that does not require physical contact with the sample. As a non-destructive technology, the device can be used to measure the viscoelastic properties of gels at rest [34].

In Figure S4, a representative MSD plot (niaprazine 0.3% *w*/*v*-loaded XG gel) is shown. It is evident that the MSD curves change over time, suggesting the viscoelastic nature of the sample due to the decreased movement of particles within the gel.

**Figure 6:**
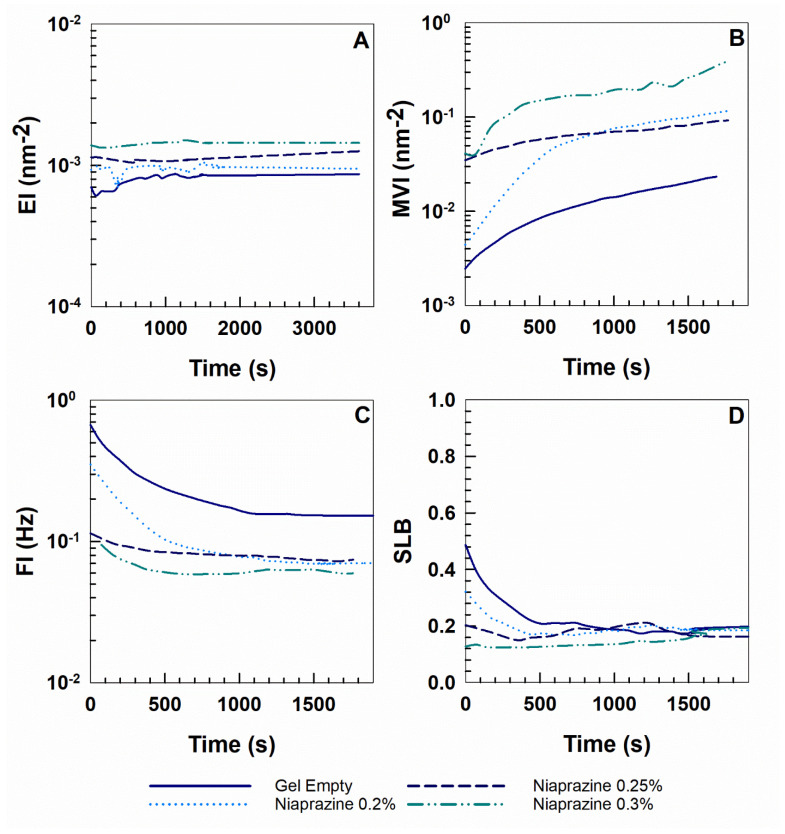
(**A**) Elasticity Index (EI), (**B**) Macroscopic Viscosity Index (MVI), (**C**) Fluidity Index (FI), and (**D**) Solid Liquid Balance (SLB) versus time curves of xanthan gum gels with various concentrations of niaprazine (0–0.3% *w*/*v*) at 25 °C.

The elasticity strength, viscosity, and fluidity of XG gels can be evaluated by EI, MVI, and FI profiles (Figure 6). EI can be calculated from the reciprocal of the height of the plateau region in the MSD curve [35]. A higher plateau value results in a lower EI, and a higher EI indicates a greater elasticity due to the particles that cover shorter distances before the network interaction [36].

MVI, which is the inverse of particle speed over long time periods, quantifies the macroscopic viscosity at zero-shear [37]. The slope of the MSD curve after the plateau is characteristic of the macroscopic viscosity of the product. Indeed, the lower this slope, the less distance the particles cross as a function of a given decorrelation time. The slower the particles move, the stronger the viscosity of the product [37].

Lastly, FI identifies the fluidity of particles in the system and is obtained as the inverse of the decorrelation time [36].

As shown in Figure 6, the EI and MVI values of the XG gels increased in a concentration-dependent manner with respect to the drug, in contrast to the FI profiles. This trend could be due to the limited motion of the particles, deriving from the increased viscosity of the sample. The motion of the particles was probably hindered by the interactions occurring within the network, leading to a reduction in their speed.

These results are consistent with those previously reported through dynamic rheology studies.

Moreover, the SLB parameter, which is the ratio between the solid-like and liquid-like behavior, was used to characterize the solid/liquid characteristics of the samples [38]. SLB is obtained from the slope of the plateau region of the MSD curves. A higher slope indicates a faster motion of the particles, suggesting a decrease in the viscosity of the sample. Conversely, a lower slope corresponds to a decrease in the motional rate of particles as they are hindered by the cage, indicating the elastic properties of the sample [39].

Generally, SLB = 0 is characteristic of pure elastic samples, 0 ˂ SLB ˂ 0.5 identifies mainly solid-like samples, while 0.5 ˂ SLB ˂ 1 indicates the predominance of a liquid-like behavior. SLB = 1 represents a sample with pure viscous properties [40].

The SLB profiles of all the investigated formulations were below 0.5, indicating a solid-like behavior of the samples, as previously demonstrated by the mechanical spectra (Figure 6).

### 2.4. Syringeability and Mucoadhesive Properties

The administration of drugs to children remains a significant challenge. The risk of a wrong dosage is higher in children than in adults because pediatric doses are based on age, weight, and body surface area [41]. It is well known that using a metal kitchen spoon to measure and administer oral medication is inaccurate due to the variability of the volume, which can lead to substantial underdosing or overdosing. Oral syringes are recommended for their superior accuracy as compared to graduated pipettes or measuring spoons [42].

**Figure 7:**
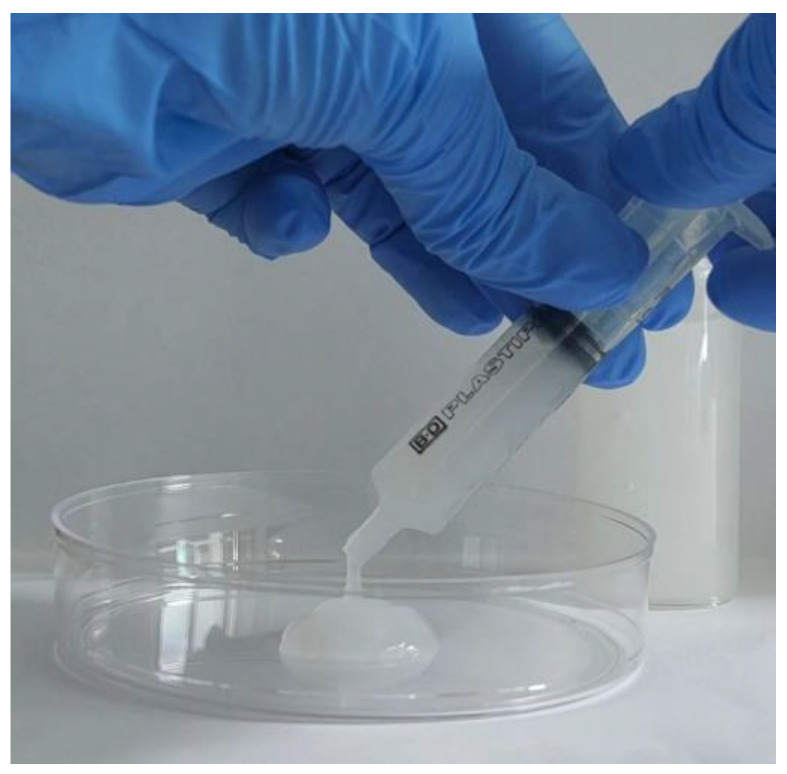
Syringeability test of niaprazine (0.3%, *w*/*v*)-loaded XG gel through a 5 mL syringe.

Therefore, the syringeability of XG gels was evaluated by a specific test. The gel with the highest drug concentration (0.3% *w*/*v*) was loaded into a 5 mL syringe and injected into a Petri dish. As shown in Figure 7 and Video S2, the formulation flowed smoothly through the syringe and retained its shape after injection. This test confirms that the proposed formulations are suitable for easy administration to a pediatric population using a syringe, allowing the modulation of the dosage as a function of body weight [15].

Another important property to consider in the development of an oral pharmaceutical formulation is its mucoadhesion. The term describes the interaction between a system and the mucous layers [43,44].

Mucoadhesive properties are highly desirable as they promote the modulation of frictional forces involved in activities such as mastication, speaking, saliva secretion, and swallowing. Mucoadhesive agents also protect drugs from degradation in the acidic environment of the gastrointestinal tract, leading to faster onset of action through direct contact with mucosal surfaces [44].

Scientific literature widely supports the use of XG as an effective mucoadhesive agent [25].

Rheological analysis is a widely used approach to assess the mucoadhesion potential of a pharmaceutical formulation. In fact, mucoadhesive delivery systems are commonly expected to exhibit a rheological response in mucin mixtures that exceeds the combined contributions of the systems and mucin individually. This phenomenon is often referred to as ’rheological synergism’, Δη (here reported as mucoadhesion index).

**Figure 8:**
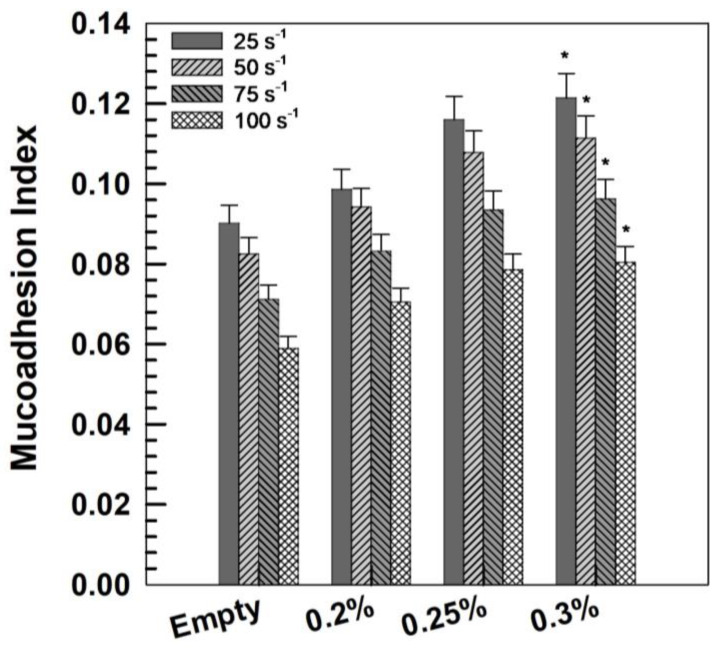
Mucoadhesion index values of niaprazine (0–0.3% *w*/*v*)-loaded XG gels measured at 37 °C as a function of the shear rate (25, 50, 75, and 100 s^−1^). * *p* < 0.01 with respect to the empty formulation.

Figure 8 shows the variation in Δη values with respect to the shear rate. All XG gels were characterized by positive mucoadhesion index values at all the investigated shear rates. These data demonstrate that the viscosity of the mixtures is higher than that of the single components or their sum, demonstrating the mucoadhesiveness of XG gels containing niaprazine.

Moreover, for each formulation, rheological synergism decreases when the shear rate increases, suggesting that low stress reduces the disentanglement and disruption of the gels [45].

It is possible to evaluate the nature of the interaction between the gel and mucin by applying a constant deformation in the frequency range between 0.1 and 10 Hz [46,47].

**Figure 9:**
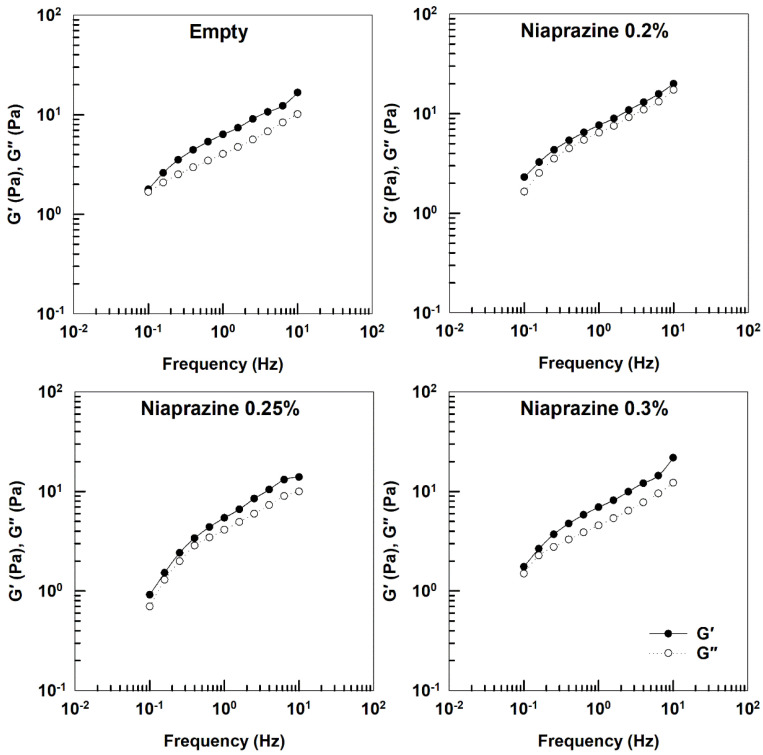
Frequency sweep measurements of xanthan gum gels/mucin mixtures at 37 °C.

The mechanical spectra (Figure 9) show that the storage modulus G′ is slightly higher than G″ in all systems, suggesting interaction with mucin and the formation of secondary chemical bonds between the gels and mucin [46,47].

This type of interaction is consistent with the trend already described in other experimental investigations focused on the use of XG solutions at the polymer concentration (1% *w*/*v*) employed in this study [47].

### 2.5. Drug Release Profiles

**Figure 10:**
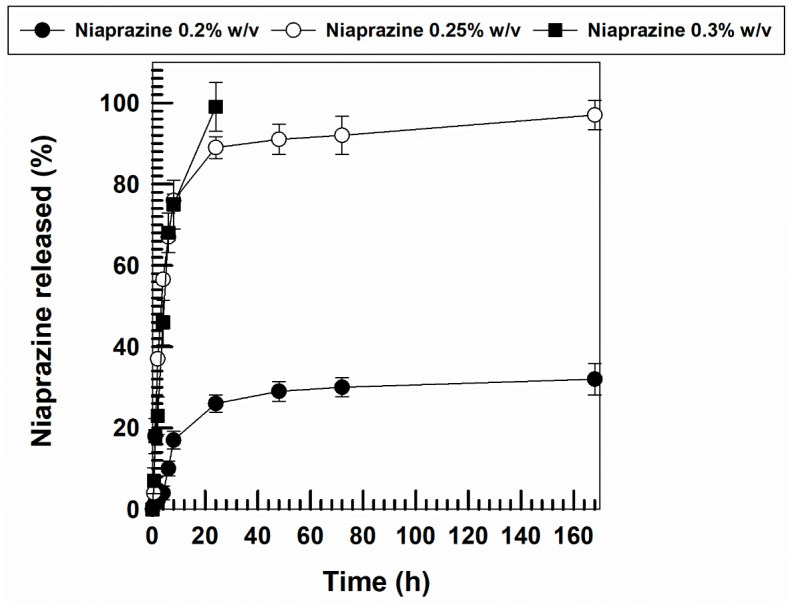
Release profiles of niaprazine (0.2–0.3% *w*/*v*) from xanthan gum-based gels. Experiments were carried out at 37 °C. Values are the average of three different experiments ± standard deviation.

In vitro drug release studies were carried out in order to evaluate the leakage kinetics of niaprazine from the XG gels. The results are reported in Figure 10.

The cumulative release of the active compound was proportional to the concentration of the molecule contained in the matrix gel. In detail, the highest drug concentration promoted the fastest release of niaprazine. An initial massive leakage was obtained in the formulation prepared with 0.25 and 0.3% *w*/*v* of drug (~80% in the first 10 h), followed by a complete release of the active compound within 24 h for the system containing the highest concentration of sedative agent. The other formulations were characterized by a constant and prolonged drug leakage over several days.

These findings support the hypothesis of a weak interaction between niaprazine and the XG network [48].

The correlation coefficients of the release kinetics demonstrate that the leakage of niaprazine follows the first-order model, confirming a drug concentration-dependent mechanism (Table 1) [15].

The release trend was confirmed when it was investigated under simulated gastric (pH 1.2) and intestinal fluids (pH 6.8), respectively, as reported in Figure S5 [49].

## 3. Conclusions

An XG gel containing niaprazine for pediatric use was prepared and analyzed in this study.

The formulations showed significant stability and desirable physical properties, such as white-transparent color and a non-sticky, non-gritty consistency [15]. XG-based systems showed weak gel behavior with excellent shear-thinning properties, facilitating flexible, easy dosing based on body weight and ease of swallowing. Moreover, their viscosity prevented drug precipitation, thereby minimizing the risk of both under- and over-dosing [15].

The mucoadhesive properties of XGs are expected to enhance the drug bioavailability, promoting a faster onset of action through direct contact with mucosal surfaces [44].

Niaprazine-loaded XG gels could be an effective oral delivery system for the sedative compound, representing a conceivable alternative to syrups and able to ensure an accurate administration dosage while improving compliance in pediatric patients.

## 4. Materials and Methods

### 4.1. Materials

Sodium benzoate, xanthan gum (XG), glycerol, ethylenediaminetetraacetic acid disodium salt (EDTA), and strawberry flavor were purchased from Farmalabor (Canosa di Puglia, Italia).

Sodium saccharin and niaprazine were provided by Galeno (Prato, Italia) and Acef S.p.A. (Piacenza, Italia), respectively. Ethanol (EtOH) was supplied by Fagron (Bologna, Italia).

For the mucoadhesive studies, dried mucin from porcine stomach type II (Merck, Milan, Italy) was used.

Deionized double-distilled water was used throughout the study. All other materials and solvents used in this investigation were of analytical grade (Carlo Erba, Milan, Italy).

### 4.2. Development of Niaprazine-Loaded Gels

#### 4.2.1. Pre-Formulation Studies and Gel Composition

Initially, studies were performed to identify a suitable solvent for solubilizing the active compound and preventing its precipitation.

Ethanol was chosen, taking into account the limits of use in the pediatric age indicated in Rowe et al.: 10% *v*/*v* for formulations designed for use by people of 12 years of age and older, 5% *v*/*v* for preparations intended for use by children aged 6–12 years of age, and 0.5% *v*/*v* for products for use by children under the age of 6 [24].

In the formulation containing the highest concentration of niaprazine (3 mg/mL), 450 mg of the drug was solubilized in 7 mL of ethanol (final volume 150 mL), obtaining a percentage of organic solvent equal to 4.6% *v*/*v*. This concentration allows the formula to remain within the 5% *v*/*v* limit, making possible its use in a larger patient population.

The next goal was to obtain a formulation with suitable rheological properties for oral administration.

Xanthan gum (XG) was selected because of its excellent flow properties and thermal stability [25]. XG is a biocompatible, biodegradable, nontoxic, and low-cost anionic polysaccharide produced by the bacteria *Xanthomonas campestris*, extensively used in the food and pharmaceutical industries [50,51]. The polysaccharide has several favorable properties for designing various forms of drug delivery systems [25]. Due to its high molecular weight and hydrogen bonding interactions, aqueous solutions of XG exhibit high intrinsic viscosity at low concentrations (1% *w*/*v*) [52]. Moreover, due to its intrinsically high stability at low pHs, XG-based systems help protect the drug from degradation in gastric fluid [25]. The other components used for the preparation of the gel and their relative concentrations and functions are summarized in Table 2.

All the measuring weights were carried out with an analytical scale Mettler Toledo^®^ equipped with LabX^®^ software (Version 13.0) to ensure data traceability and a precise and fully reproducible work process [53,54].

#### 4.2.2. Preparation of Xanthan Gum Gels by IKA^®^ LR 1000 Control Reactor

XG-based gels were prepared using an IKA^®^ LR reactor (1000 control, Staufen, Germany). A T25 Ultra-Turrax dispersing tool equipped with an S25 KV-25 F rotor head (IKA, Staufen, Germany) was employed to mix the components at a speed of 20K rpm (Figure S6).

Three concentrations of niaprazine were employed: 0.20% *w*/*v*, 0.25% *w*/*v*, and 0.30% *w*/*v*.

The XG was dispersed in glycerol, and then, an appropriate amount of water and all the hydrophilic components (i.e., sodium benzoate, sodium EDTA, and saccharin) were added to the reactor vessel at 25 °C for 30 min at a stirring rate of 100 rpm. When all the components were dissolved, the XG dispersed in glycerol was gradually added. During gel formation, niaprazine, previously dissolved in ethanol, was poured into the system. Finally, the flavoring agent was added to improve the palatability of the product.

The resulting formulation was transferred into a 500 mL graduated cylinder and adjusted to the final volume.

These operations were carried out in a Good Manufacturing Practice (GMP)-approved laboratory according to the international guidelines for the preparation of medicinal products [55].

### 4.3. Characterization of Niaprazine Gel

#### 4.3.1. Evaluation of Stability Profiles by Multiple Light Scattering Technique

Physical stability measurements of the resulting XG-based gels were performed using a Turbiscan^®^ Lab Expert analyzer (Formulaction, L’Union, France) [56].

A total of 20 mL of each gel was placed into borosilicated cylindrical glass vials equipped with stoppers and scanned from bottom to top for a total of 1 h. The resulting data were processed by a Turby Soft 2.0 software program and reported as Turbiscan Stability Index (TSI) and delta backscattering (ΔBS) vs. time and temperature, as previously reported [57].

The TSI is a statistical parameter used to evaluate the variations of the backscattering (BS) and transmission (T) of a sample over time. The TSI is useful for investigating adverse physical phenomena, such as sedimentation, creaming, or flocculation, providing information about the stability of a formulation [56].

#### 4.3.2. Macroscopic Properties

The macroscopic features of each formulation were evaluated through visual observation, assessing homogeneity and measuring pH.

Niaprazine gels were visually observed to assess their clarity, texture, and presence of extraneous particles. The texture of the prepared gels was assessed by rubbing them between two fingers to determine their grittiness and stickiness [15]. Any occurrence of syneresis phenomena was also observed after storing the systems at room temperature (25 ± 2 °C) for 24 h and 90 days [15].

A homogeneity test was performed to verify the uniformity of the distribution of the niaprazine throughout the gel. A 1 mL sample was taken from different regions of the gel containing the higher drug concentration (0.3% *w*/*v*) (upper, middle, and lower), dissolved in 100 mL of ethanolic solution 70:30 *v*/*v* and subjected to continuous shaking for 4 h. Subsequently, the samples were filtrated and analyzed using a spectrophotometer (Perkin-Elmer GmbH, Uberlingen, Germany) [58].

The pH was evaluated using a digital pH meter, ensuring that the pH of the formulations was within the desired range (5–8) [24].

#### 4.3.3. Rheological Studies

Shear viscometry and oscillation measurements were performed using a Kinexus^®^ Pro rotational rheometer (Malvern Panalytical Ltd., Spectris plc, Worcestershire, England) with cone-plate geometry (40 mm; angle of 2°) and equipped with a temperature-controlling Peltier unit and a solvent trap to avoid evaporation during analyses [56].

Flow curves were determined over shear rates ranging from 0.1 to 100 s^−1^ at 25 °C and 37 °C [40].

An amplitude sweep measurement was performed to determine the shear-independent plateau of the linear viscoelastic region (LVR). Subsequent frequency sweep measurements were carried out at stress values in the shear-independent plateau (1 Pa) over the frequency range from 10 to 0.1 Hz to determine the storage or elastic modulus (G′), the loss or viscous modulus (G″), and the phase angle δ [39,56].

The samples were carefully placed onto the inferior plate and allowed to equilibrate for at least 5 min before each analysis.

The microrheological properties of niaprazine-loaded gels were investigated through diffusive wave spectroscopy (DWS) analysis using a Rheolaser^®^ Master apparatus (Formulaction, Toulouse, France) at 25 °C, as previously reported [40,59].

Briefly, the particles in the formulation are used to measure local deformations by assessing their Brownian motion and the mean square displacement (MSD), which is the average distance traveled by the particles in the medium [40].

The parameter reflects the freedom of motion of particles and is directly linked to the decorrelation time, where the slope correlates with the viscosity of the product. In viscoelastic materials, particles are confined within a “cage” formed by interactions with other particles, imparting elasticity to the product. Depending on the decorrelation time scale observed, three distinct regions could be identified. At very short decorrelation times, particles move freely within the cage, leading to a linear increase in MSD. Intermediate decorrelation times demonstrate an interaction between particles and the structure of a “cage”, slowing their motion and causing the MSD slope to plateau, demonstrating a certain elasticity. Long decorrelation times indicate that particles escape from the “cage”, resulting in an initially increasing MSD slope that eventually grows linearly with decorrelation time, reflecting the macroscopic viscosity of the product [40].

Elasticity index (EI), macroscopic viscosity index (MVI), fluidity index (FI), and solid–liquid balance (SLB) values were calculated from MSD curves using Rheosoft Master 1.4.0 software [40].

#### 4.3.4. Syringeability

The syringeability of the XG-based systems containing niaprazine (0.3% *w*/*v*) was investigated at room temperature using a 5 mL syringe. If the system flowed through the syringe quickly, it was defined as “passing”, while if it struggled or was difficult to pass, it was classified as “failed” [60].

#### 4.3.5. Mucoadhesive Properties

The mucoadhesive properties of the gels were investigated using the method reported by Račić et al. [46]. This method evaluates the “rheological synergism” between the pharmaceutical system and mucin [46].

A mucin dispersion (20% *w*/*w*) was prepared by dispersing porcine mucin in the required amount of purified water under mechanical stirring. The tested formulations were mixed with the freshly prepared dispersion or purified water at a ratio of 1:1 (*w*/*w*).

Viscosity measurements were performed at 37 °C using a rotational rheometer with shear rates ranging from 0.1 to 100 s^−1^ (Section 4.3.3). After measurements, the viscosity values at different shear rates (25, 50, 75, 100 s^−1^) were used to calculate the Mucoadhesion Index Δη (or “rheological synergism”) from the equation:

Δη = η_mix_ − (η_gel_ + η_mucin_), (1)

where η_mix_, η_gel_, and η_mucin_ are the apparent viscosity (Pa s) of the mixture of XG gels and mucin dispersion, XG gels and the mucin dispersion, respectively.

For a mucoadhesive formulation, η_mix_ was higher than η_gel_ + η_mucin_ due to the interaction occurring between the polymeric system and mucin, resulting in positive Δη values.

In addition, oscillatory rheological analyses were carried out in order to identify the possible nature of the interaction between the XG-based systems and the mucin [46,61].

For this purpose, a frequency sweep test was performed on the mucin/gel mixtures as previously described (Section 4.3.3).

There were three possible results: (i) G′ >> G″ for chemically cross-linked systems, (ii) G′ > G″ for systems connected by secondary bonds, and (iii) G′ ≤ G″ for physically cross-linked polymeric systems [46,47].

#### 4.3.6. In Vitro Drug Release and Kinetic Data Modeling

The release kinetics of niaprazine from XG gels were investigated by means of the dialysis technique [62]. In detail, 1 mL of each sample was placed into a dialysis bag (cut-off of 10–12 kDa, Spectrum Laboratories Inc., Eindhoven, The Netherlands) and sealed at both ends with clips. The dialysis bag was immersed in 100 mL release medium (H_2_O/EtOH 70:30 *v*/*v* in order to operate under sink conditions) and kept at 37 ± 1 °C under moderate agitation. At fixed time points, 1 mL of the receptor fluid was collected and replaced by fresh [62].

The amount of drug released over time was calculated by UV-vis spectrophotometry using the equation reported below:

Release (%) = Drug_rel_/Drug_load_ × 100, (2)

where Drug_rel_ is the amount of released drug and Drug_load_ is the amount of loaded niaprazine.

In order to investigate the mechanism of the drug release, the resulting data were analyzed using various mathematical models (Table 3). Based on the R^2^ value, the best-fitted model was selected [15,63].

Moreover, the release kinetics of niaprazine from xanthan gum-based gels were investigated under simulated gastric (pH 1.2) and intestinal fluids (pH 6.8), as previously reported [49].

### 4.4. Statistical Analysis

Statistical evaluation was performed using a one-way analysis of variance test (ANOVA) considering a *p*-value of <0.05 significant.

Data were expressed as mean and standard deviation (mean ± SD) of separate experiments.

## Figures and Tables

**Table 1:** Correlation coefficient (r^2^) of release kinetics of niaprazine from xanthan gum-based gels according to various mathematical models.

Niaprazine (% *w*/*v*)	Zero Order	First Order	Higuchi	Korsmeyer–Peppas	Hixson–Crowell
0.2	0.906	0.926	0.769	0.718	0.915
0.25	0.926	0.989	0.966	0.943	0.973
0.3	0.976	0.988	0.955	0.899	0.969

**Table 2:** Composition of Xanthan Gum-based Oral Gels.

Excipients	Function	% *w*/*v*
Ethanol	Solvent for Drug Solubilization	4.60 ^1^
Xanthan Gum	Gelling and Stabilizing Agent	1.00
Glycerol	Solvent and Viscosity-augmenting Agent	12.3
Sodium Benzoate	Preservative	0.19
Sodium EDTA	Chelating Agent	0.10
Sodium Saccharin	Sweetening Agent	0.07
Strawberry Flavor	Flavoring Agent—enhances patient acceptance	0.10
Purified Water	Solvent	up to 500 mL

^1^ % *v*/*v*.

**Table 3:** Kinetic models used for the analysis of release profiles.

Kinetic Model	Equation	In Vitro Parameters
Zero-order	*F_t_* = *K*_0_*t*	*F_t_*: amount of drug released at time *t*; *K*_0_: apparent rate constant
First-order	ln(1 − *F*) = −*K*_1_*t*	*F*: quantity of drug released at time *t*; *K*_1_: first-order release rate constant
Higuchi	*F* = *K*_2_*t*^1/2^	*F*: total of drug released at time *t*; *K*_2_: Higuchi constant
Korsmeyer–Peppas	*M_t_*/*M_¥_* = *K*_3_*t*^n^	*M_t_*: water mass at time *t*; *M_¥_*: water mass obtained at equilibrium; *K*_3_: constant incorporating structural and geometric characteristics of the drug-dosage form; *n*: release exponent indicating the drug-release mechanism

## Data Availability

The raw data supporting the conclusions of this article will be made available by the authors on request.

## Supplementary Materials

The following supporting information can be downloaded at https://www.mdpi.com/article/10.3390/gels11020101/s1, Figure S1: Delta backscattering profiles of xanthan gum gels containing various concentrations of niaprazine (0–0.30% *w*/*v*) as a function of the temperature and incubation time. Analyses were performed at 25 °C (left) and 37 °C (right); Figure S2: Xanthan gum-based gels containing various concentrations of niaprazine; Video S1: Stickiness test of niaprazine-loaded xanthan gum gels; Table S1: Homogeneity of niaprazine 0.3% *w*/*v*-loaded gel; Figure S3: (A) Chemical Structure of Xanthan gum. (B) Schematic representation of the structural transition mechanism of xanthan gum; Figure S4: Mean square displacement (MSD) curves of xanthan gum gel containing niaprazine (0.3% *w*/*v*) as a function of the decorrelation time at 25 °C. The different colors of the curves correspond to a single scan performed throughout the analysis; Video S2: Syringeability test of niaprazine-loaded xanthan gum gels. Figure S5. Release profiles of niaprazine (0.2–0.3 *w*/*v*) from xanthan gum-based gels investigated under simulated gastric (pH 1.2) and intestinal fluids (pH 6.8), respectively. Values represent mean of five different experiments ± standard deviation; Figure S6: The IKA^®^ LR 1000 reactor (Staufen, Germany) equipped with the T25 Ultra-Turrax dispersing tool (IKA, Staufen, Germany) used for the preparation of niaprazine-loaded XG gels.

## References

[1] Crommelin D.J.A., Bouwman-Boer Y. (2016). Pharmacy preparations: Back in the limelight? Pharmacists make up your mind!. Int. J. Pharm..

[2] Brion F., Nunn A.J., Rieutord A. (2003). Extemporaneous (magistral) preparation of oral medicines for children in European hospitals. Acta Paediatr..

[3] Minghetti P., Pantano D., Gennari C.G.M., Casiraghi A. (2014). Regulatory framework of pharmaceutical compounding and actual developments of legislation in Europe. Health Policy.

[4] Zanon D., Musazzi U.M., Cirino M., Bennati G., Casiraghi A., Maximova N., Barbi E., Minghetti P. (2023). Cases of drug repositioning in children’s orphan drugs: Licenced drugs versus unlicenced magistral preparations. J. Drug Deliv. Sci. Technol..

[5] Reed M.D., Findling R.L. (2002). Overview of current management of sleep disturbances in children: I—Pharmacotherapy. Curr. Ther. Res..

[6] Montanari G., Schiaulini P., Covre A., Steffan A., Furlanut M. (1992). Niaprazine vs chlordesmethyldiazepam in sleep disturbances in pediatric outpatients. Pharmacol. Res..

[7] Younus M., Labellarte M.J. (2002). Insomnia in Children. Pediatr. Drugs.

[8] Ottaviano S., Giannotti F., Cortesi F. (1991). The effect of niaprazine on some common sleep disorders in children. Child’s Nerv. Syst..

[9] Scherman D., Hamon M., Gozlan H., Henry J.-P., Lesage A., Masson M., Rumigny J.F. (1988). Molecular pharmacology of niaprazine. Prog. Neuro-Psychopharmacol. Biol. Psychiatry.

[10] Keane P.E., Benedetti M.S., Dow J. (1982). The effect of niaprazine on the turnover of 5-hydroxytryptamine in the rat brain. Neuropharmacology.

[11] Esposito D., Belli A., Ferri R., Bruni O. (2020). Sleeping without Prescription: Management of Sleep Disorders in Children with Autism with Non-Pharmacological Interventions and Over-the-Counter Treatments. Brain Sci..

[12] Klingmann V., Spomer N., Lerch C., Stoltenberg I., Frömke C., Bosse H.M., Breitkreutz J., Meissner T. (2013). Favorable Acceptance of Mini-Tablets Compared with Syrup: A Randomized Controlled Trial in Infants and Preschool Children. J. Pediatr..

[13] Al Humaid J. (2018). Sweetener content and cariogenic potential of pediatric oral medications: A literature. Int. J. Health Sci..

[14] Ogbonna J.D.N., Cunha E., Attama A.A., Ofokansi K.C., Ferreira H., Pinto S., Gomes J., Marx Í.M.G., Peres A.M., Lobo J.M. (2022). Overcoming Challenges in Pediatric Formulation with a Patient-Centric Design Approach: A Proof-of-Concept Study on the Design of an Oral Solution of a Bitter Drug. Pharmaceuticals.

[15] Mawazi S.M., Al-Mahmood S.M.A., Chatterjee B., Hadi H.A., Doolaanea A.A. (2019). Carbamazepine Gel Formulation as a Sustained Release Epilepsy Medication for Pediatric Use. Pharmaceutics.

[16] Layek B., Mandal S. (2020). Natural polysaccharides for controlled delivery of oral therapeutics: A recent update. Carbohydr. Polym..

[17] Petri D.F.S. (2015). Xanthan gum: A versatile biopolymer for biomedical and technological applications. J. Appl. Polym. Sci..

[18] Kumar P., Kumar B., Gihar S., Kumar D. (2024). Review on emerging trends and challenges in the modification of xanthan gum for various applications. Carbohydr. Res..

[19] Layek B. (2024). A Comprehensive Review of Xanthan Gum-Based Oral Drug Delivery Systems. Int. J. Mol. Sci..

[20] Belayneh A., Tessema Z. (2021). A Systematic Review of the Stability of Extemporaneous Pediatric Oral Formulations. Sci. World J..

[21] Gagliardi A., Ambrosio N., Voci S., Salvatici M.C., Fresta M., Cosco D. (2022). Easy preparation, characterization and cytotoxic investigation of 5-Fluorouracil-loaded zein/sericin nanoblends. J. Mol. Liq..

[22] Fan W., Shi Y., Hu Y., Zhang J., Liu W. (2024). Effects of the Combination of Protein in the Internal Aqueous Phase and Polyglycerol Polyricinoleate on the Stability of Water-In-Oil-In-Water Emulsions Co-Encapsulating Crocin and Quercetin. Foods.

[23] Cosco D., Paolino D., Maiuolo J., Marzio L.D., Carafa M., Ventura C.A., Fresta M. (2015). Ultradeformable liposomes as multidrug carrier of resveratrol and 5-fluorouracil for their topical delivery. Int. J. Pharm..

[24] Rowe R.C., Sheskey P., Quinn M. (2009). Handbook of Pharmaceutical Excipients.

[25] Jadav M., Pooja D., Adams D.J., Kulhari H. (2023). Advances in Xanthan Gum-Based Systems for the Delivery of Therapeutic Agents. Pharmaceutics.

[26] Liu Z., Yao P. (2015). Injectable thermo-responsive hydrogel composed of xanthan gum and methylcellulose double networks with shear-thinning property. Carbohydr. Polym..

[27] Song K.-W., Kim Y.-S., Chang G.-S. (2006). Rheology of concentrated xanthan gum solutions: Steady shear flow behavior. Fibers Polym..

[28] Kennedy J.R.M., Kent K.E., Brown J.R. (2015). Rheology of dispersions of xanthan gum, locust bean gum and mixed biopolymer gel with silicon dioxide nanoparticles. Mater. Sci. Eng. C.

[29] Song J., Li J., Zhong J., Guo Z., Xu J., Chen X., Qiu M., Lin J., Han L., Zhang D. (2024). An oral gel suitable for swallowing: The effect of micronization on the gel properties and microstructure of κ-carrageenan. Int. J. Biol. Macromol..

[30] Hou Y., Sun Y., Zhang P., Wang H., Tan M. (2023). Development and characterization of emulsion gels prepared via gliadin-based colloidal particles and gellan gum with tunable rheological properties for 3D printed dysphagia diet. Int. J. Biol. Macromol..

[31] Xu L., Xu G., Liu T., Chen Y., Gong H. (2013). The comparison of rheological properties of aqueous welan gum and xanthan gum solutions. Carbohydr. Polym..

[32] Martín-Alfonso J.E., Cuadri A.A., Berta M., Stading M. (2018). Relation between concentration and shear-extensional rheology properties of xanthan and guar gum solutions. Carbohydr. Polym..

[33] Li X., Harding S.E., Wolf B., Yakubov G.E. (2022). Instrumental characterization of xanthan gum and scleroglucan solutions: Comparison of rotational rheometry, capillary breakup extensional rheometry and soft-contact tribology. Food Hydrocoll..

[34] Mao Y., Nielsen P., Ali J. (2022). Passive and Active Microrheology for Biomedical Systems. Front. Bioeng. Biotechnol..

[35] Cai W., Hu T., Huang Q. (2022). Rheological properties and critical concentrations of a hyperbranched polysaccharide from *Lignosus rhinocerotis* sclerotia. Int. J. Biol. Macromol..

[36] Yue M., Huang M., Zhu Z., Huang T., Huang M. (2022). Effect of ultrasound assisted emulsification in the production of Pickering emulsion formulated with chitosan self-assembled particles: Stability, macro, and micro rheological properties. LWT.

[37] Su J., Guo Q., Chen Y., Dong W., Mao L., Gao Y., Yuan F. (2020). Characterization and formation mechanism of lutein pickering emulsion gels stabilized by β-lactoglobulin-gum arabic composite colloidal nanoparticles. Food Hydrocoll..

[38] Sun C., Wu T., Liu R., Liang B., Tian Z., Zhang E., Zhang M. (2015). Effects of superfine grinding and microparticulation on the surface hydrophobicity of whey protein concentrate and its relation to emulsions stability. Food Hydrocoll..

[39] Gagliardi A., Froiio F., Salvatici M.C., Paolino D., Fresta M., Cosco D. (2020). Characterization and refinement of zein-based gels. Food Hydrocoll..

[40] Giuliano E., Fresta M., Cosco D. (2021). Development and characterization of poloxamine 908-hydrogels for potential pharmaceutical applications. J. Mol. Liq..

[41] Venables R., Stirling H., Batchelor H., Marriott J. (2015). Problems with oral formulations prescribed to children: A focus group study of healthcare professionals. Int. J. Clin. Pharm..

[42] Batchelor H.K., Marriott J.F. (2015). Formulations for children: Problems and solutions. Br. J. Clin. Pharmacol..

[43] Giuliano E., Paolino D., Fresta M., Cosco D. (2018). Mucosal applications of poloxamer 407-based hydrogels: An overview. Pharmaceutics.

[44] Kumar A., Naik P.K., Pradhan D., Ghosh G., Rath G. (2020). Mucoadhesive formulations: Innovations, merits, drawbacks, and future outlook. Pharm. Dev. Technol..

[45] Pereira M., Silva F.C., Simões S., Ribeiro H.M., Almeida A.J., Marto J. (2022). Innovative, sugar-free oral hydrogel as a co-administrative vehicle for pediatrics: A strategy to enhance patient compliance. AAPS PharmSciTech.

[46] Račić A., Dukovski B.J., Lovrić J., Dobričić V., Vučen S., Micov A., Stepanović-Petrović R., Tomić M., Pecikoza U., Bajac J. (2024). Synergism of polysaccharide polymers in antihistamine eye drops: Influence on physicochemical properties and in vivo efficacy. Int. J. Pharm..

[47] Ceulemans J., Vinckier I., Ludwig A. (2002). The use of xanthan gum in an ophthalmic liquid dosage form: Rheological characterization of the interaction with mucin. J. Pharm. Sci..

[48] Abu-Huwaij R., Obaidat R.M., Sweidan K., Al-Hiari Y. (2011). Formulation and In Vitro Evaluation of Xanthan Gum or Carbopol 934-Based Mucoadhesive Patches, Loaded with Nicotine. AAPS PharmSciTech.

[49] Voci S., Gagliardi A., Giuliano E., Salvatici M.C., Procopio A., Cosco D. (2024). In Vitro Mucoadhesive Features of Gliadin Nanoparticles Containing Thiamine Hydrochloride. Pharmaceutics.

[50] Ćirić A., Budinčić J.M., Medarević Đ., Dobričić V., Rmandić M., Barudžija T., Malenović A., Petrović L., Djekic L. (2022). Evaluation of chitosan/xanthan gum polyelectrolyte complexes potential for pH-dependent oral delivery of escin. Int. J. Biol. Macromol..

[51] Djekic L., Ćirić A., Jana S., Jana S. (2022). Chapter 2—Micro- and nanoscale drug delivery systems based on xanthan gum hydrogels. Micro and Nano Technologies.

[52] Patel J., Maji B., Moorthy N.S.H.N., Maiti S. (2020). Xanthan gum derivatives: Review of synthesis, properties and diverse applications. RSC Adv..

[53] Santos P., Watkinson A.C., Hadgraft J., Lane M.E. (2011). Enhanced permeation of fentanyl from supersaturated solutions in a model membrane. Int. J. Pharm..

[54] Wilent S. (2011). Minimizing Weight Measurement Uncertainty. Genet. Eng. Biotechnol. News.

[55] Domingues C., Jarak I., Veiga F., Dourado M., Figueiras A. (2023). Pediatric Drug Development: Reviewing Challenges and Opportunities by Tracking Innovative Therapies. Pharmaceutics.

[56] Giuliano E., Paolino D., Cristiano M.C., Fresta M., Cosco D. (2020). Rutin-Loaded Poloxamer 407-Based Hydrogels for In Situ Administration: Stability Profiles and Rheological Properties. Nanomaterials.

[57] Gagliardi A., Voci S., Giuliano E., Salvatici M.C., Celano M., Fresta M., Cosco D. (2021). Phospholipid/zein hybrid nanoparticles as promising carriers for the protection and delivery of all-trans retinoic acid. Mater. Sci. Eng. C.

[58] Garud N., Garud A. (2012). Preparation and in-vitro evaluation of metformin microspheres using non-aqueous solvent evaporation technique. Trop. J. Pharm. Res..

[59] Gagliardi A., Giuliano E., Voci S., Costa N., Bulotta S., Salvatici M.C., Ambrosio N., Paolino D., Siddique F., Majid M. (2024). Rutin-loaded zein gel as a green biocompatible formulation for wound healing application. Int. J. Biol. Macromol..

[60] Sanjana A., Ahmed M.G., Gowda B.H.J. (2021). Preparation and evaluation of in-situ gels containing hydrocortisone for the treatment of aphthous ulcer. J. Oral Biol. Craniofacial Res..

[61] Salzillo R., Schiraldi C., Corsuto L., D’Agostino A., Filosa R., De Rosa M., La Gatta A. (2016). Optimization of hyaluronan-based eye drop formulations. Carbohydr. Polym..

[62] Gagliardi A., Bonacci S., Paolino D., Celia C., Procopio A., Fresta M., Cosco D. (2019). Paclitaxel-loaded sodium deoxycholate-stabilized zein nanoparticles: Characterization and in vitro cytotoxicity. Heliyon.

[63] Kalyani P., Khandelwal M. (2024). Drug release kinetics from in-situ modulated agar/chitosan-bacterial cellulose patches for differently soluble drugs. Int. J. Biol. Macromol..

