# Supplementary material for: Development and Characterization of Niaprazine-Loaded Xanthan Gum-Based Gel for Oral Administration
Source: Gels. 2025 Feb 1;11(2):101. doi: 10.3390/gels11020101 (PMC11854669; doi:10.3390/gels11020101)
Supplement: Supplementary file 1 [file gels-11-00101-s001.zip › gels-3398367-Supplementary material - proofread for author.pdf]

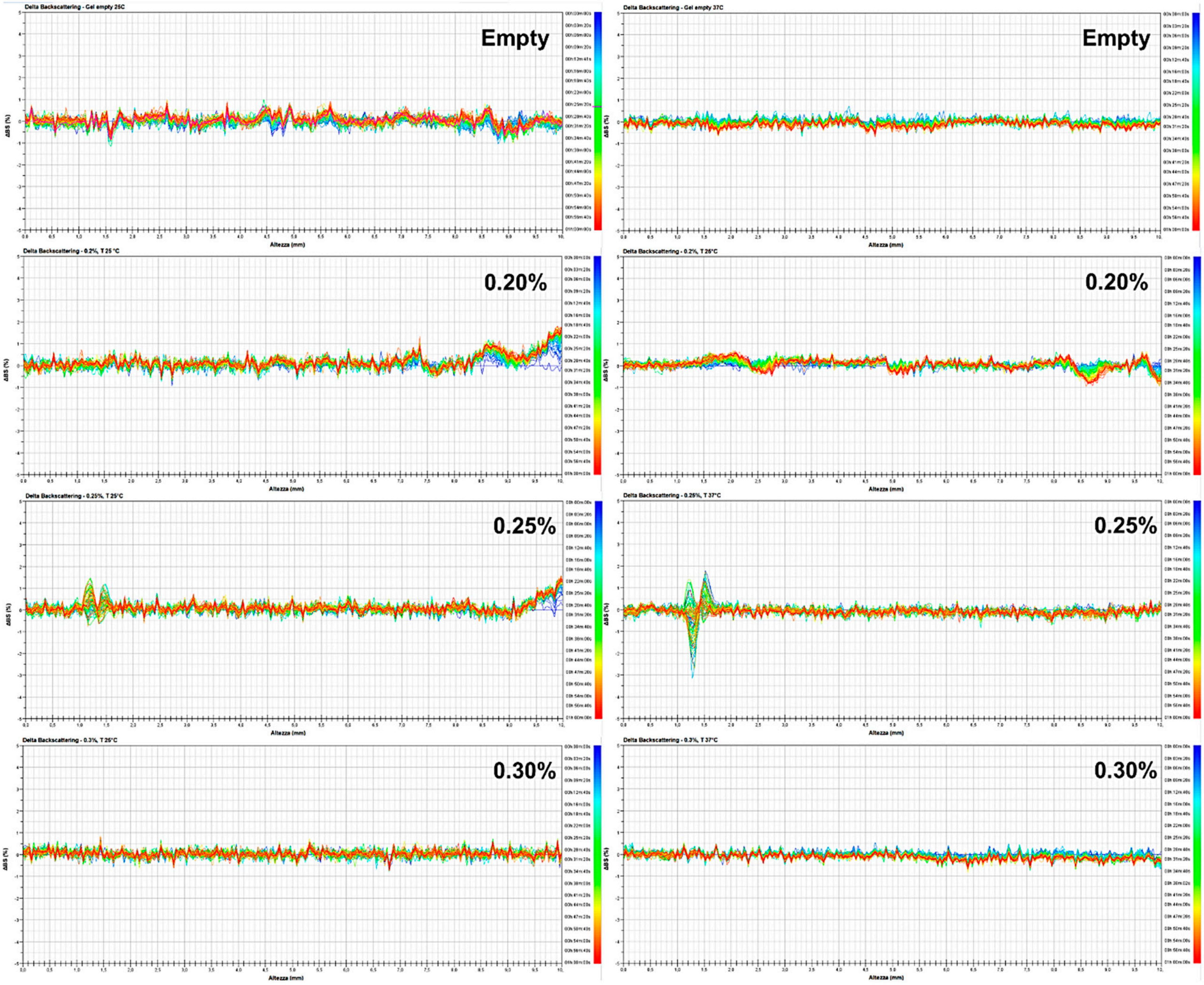

Figure S1. Delta backscattering profiles of xanthan gum gels containing various concentrations of niaprazine (0-0.30% w/v) as a function of the temperature and incubation time. Analyses were performed at 25 °C (left) and 37 °C (right).

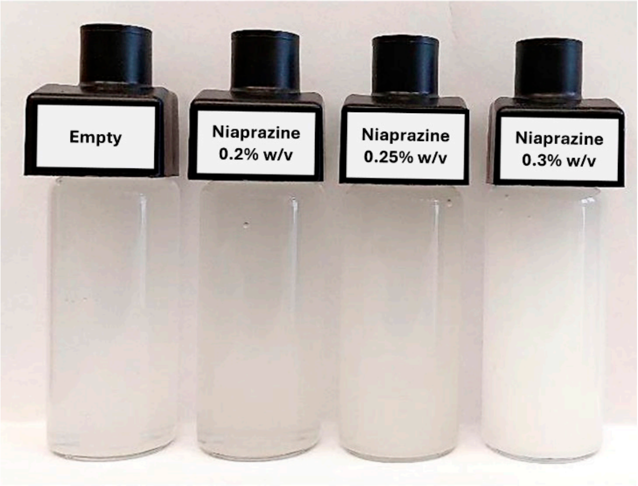

Figure S2. Xanthan gum-based gels containing various concentrations of niaprazine.

Table S1. Homogeneity of niaprazine 0.3% w/v-loaded gel.

Sample Location	% Niaprazine
Top	99.9
Middle	99.7
Bottom	99.8

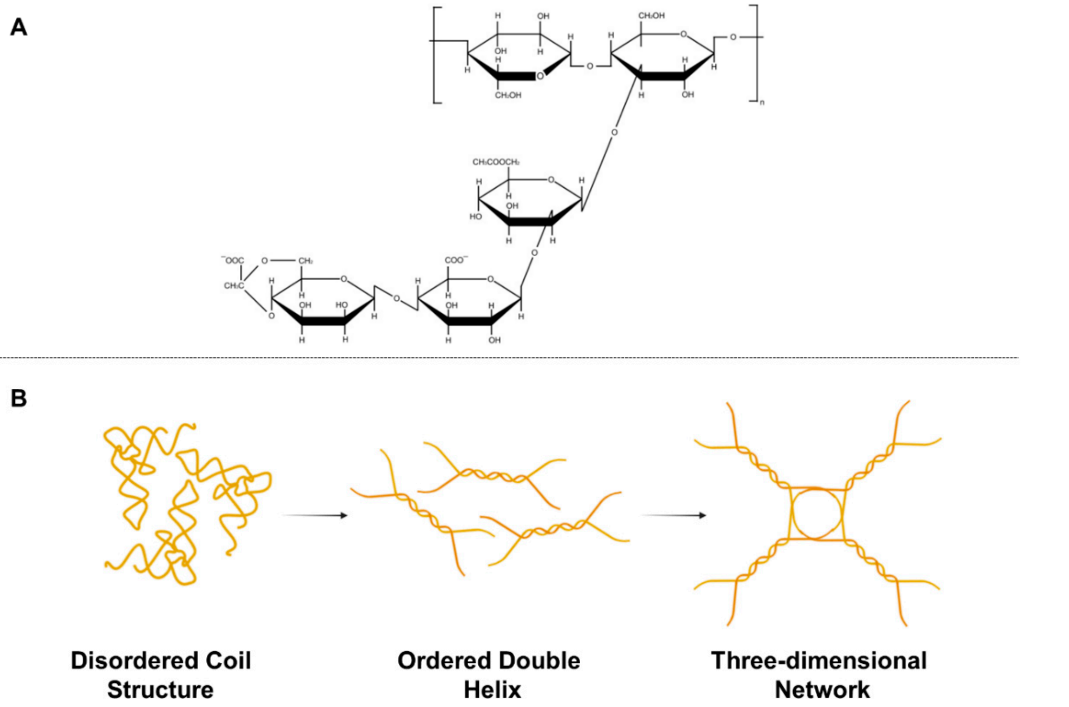

Figure S3. (A) Chemical Structure of Xanthan Gum. (B) Schematic representation of the structural transition mechanism of xanthan gum.

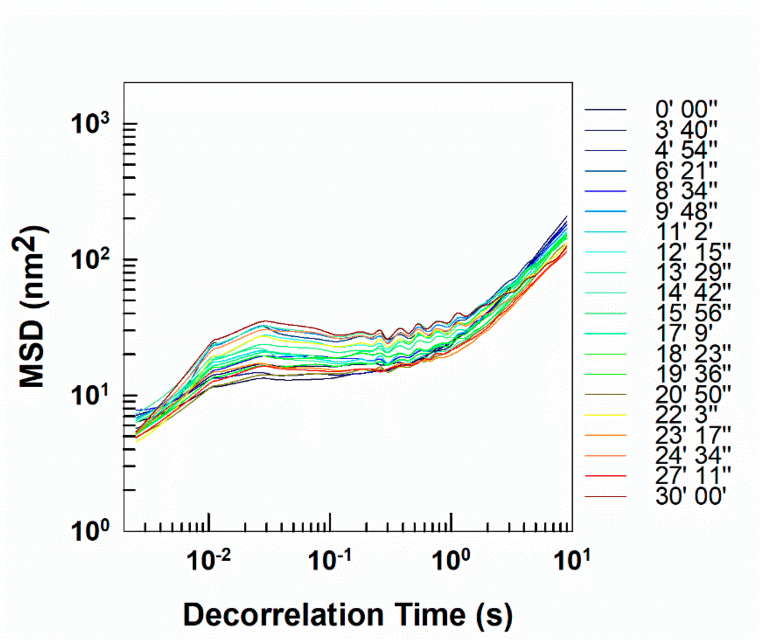

Figure S4. Mean square displacement (MSD) curves of xanthan gum gel containing niaprazine (0.3% w/v) as a function of the decorrelation time at 25 °C. The different colors of the curves correspond to a single scan performed throughout the analysis.

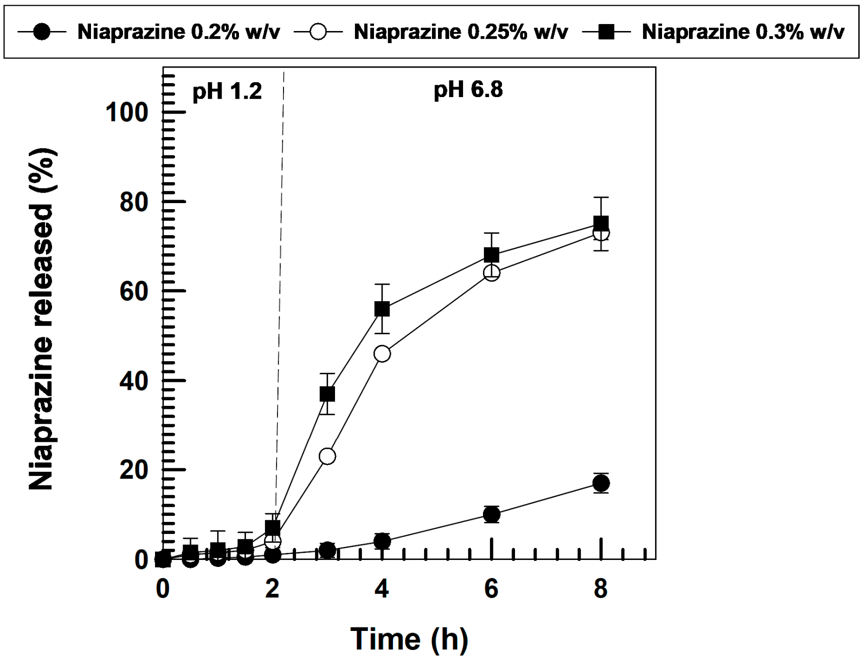

Figure S5. Release profiles of niaprazine (0.2–0.3 %w/v) from xanthan gum-based gels investigated under simulated gastric (pH 1.2) and intestinal fluids (pH 6.8), respectively. Values represent mean of five different experiments ± standard deviation.

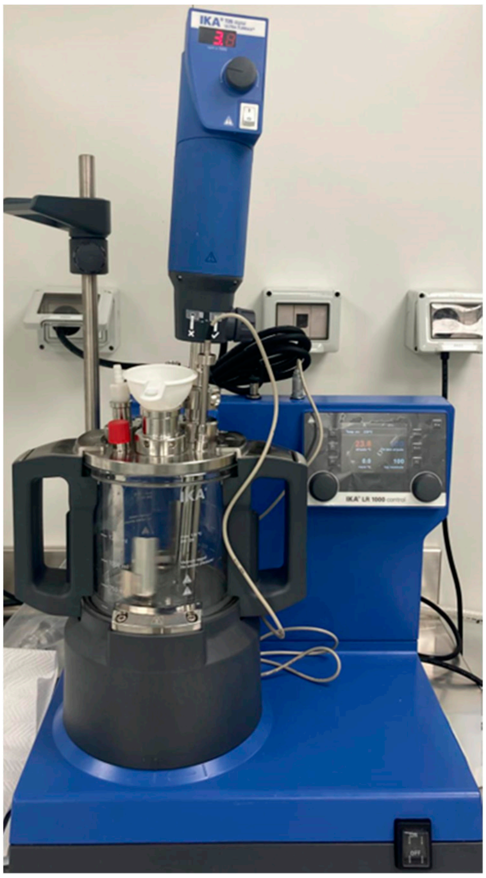

Figure S6. The IKA® LR 1000 reactor (Staufen, Germany) equipped with the T25 Ultra-Turrax dispersing tool (IKA, Staufen, Germany) used for the preparation of niaprazine-loaded XG gels.
